# A Novel Low-Risk Germline Variant in the SH2 Domain of the SRC Gene Affects Multiple Pathways in Familial Colorectal Cancer

**DOI:** 10.3390/jpm11040262

**Published:** 2021-04-01

**Authors:** Diamanto Skopelitou, Beiping Miao, Aayushi Srivastava, Abhishek Kumar, Magdalena Kuświk, Dagmara Dymerska, Nagarajan Paramasivam, Matthias Schlesner, Jan Lubiński, Kari Hemminki, Asta Försti, Obul Reddy Bandapalli

**Affiliations:** 1 Molecular Genetic Epidemiology, German Cancer Research Center (DKFZ), 69120 Heidelberg, Germany; mando.skopelitou@yahoo.de (D.S.); b.miao@kitz-heidelberg.de (B.M.); srivastava.aayushi97@gmail.com (A.S.); k.hemminki@dkfz.de (K.H.); a.foersti@kitz-heidelberg.de (A.F.); 2 Hopp Children’s Cancer Center (KiTZ), 69120 Heidelberg, Germany; 3 Division of Pediatric Neurooncology, German Cancer Research Center (DKFZ) and German Cancer Consortium (DKTK), 69120 Heidelberg, Germany; 4 Medical Faculty Heidelberg, Heidelberg University, 69120 Heidelberg, Germany; 5 Institute of Bioinformatics, International Technology Park, Bangalore 56066, India; abhishek@ibioinformatics.org; 6 Department of Genetics and Pathology, Pomeranian Medical University, 71252 Szczecin, Poland; magdalenakuswik@gmail.com (M.K.); dymerska@pum.edu.pl (D.D.); lubinski@pum.edu.pl (J.L.); 7 Computational Oncology, Molecular Diagnostics Program, National Center for Tumor Diseases (NCT), 69120 Heidelberg, Germany; n.paramasivam@dkfz.de; 8 Bioinformatics and Omics Data Analytics, German Cancer Research Center (DKFZ), 69120 Heidelberg, Germany; m.schlesner@dkfz-heidelberg.de; 9 Cancer Epidemiology, German Cancer Research Center (DKFZ), 69120 Heidelberg, Germany; 10 Faculty of Medicine and Biomedical Center in Pilsen, Charles University in Prague, 30605 Pilsen, Czech Republic

**Keywords:** familial colorectal cancer, SRC, germline variant, whole genome sequencing

## Abstract

Colorectal cancer (CRC) shows one of the largest proportions of familial cases among different malignancies, but only 5–10% of all CRC cases are linked to mutations in established predisposition genes. Thus, familial CRC constitutes a promising target for the identification of novel, high- to moderate-penetrance germline variants underlying cancer susceptibility by next generation sequencing. In this study, we performed whole genome sequencing on three members of a family with CRC aggregation. Subsequent integrative in silico analysis using our in-house developed variant prioritization pipeline resulted in the identification of a novel germline missense variant in the *SRC* gene (V177M), a proto-oncogene highly upregulated in CRC. Functional validation experiments in HT-29 cells showed that introduction of *SRC^V177M^* resulted in increased cell proliferation and enhanced protein expression of phospho-SRC (Y419), a potential marker for SRC activity. Upregulation of *paxillin*, *β-Catenin,* and *STAT3* mRNA levels, increased levels of phospho-ERK, CREB, and CCND1 proteins and downregulation of the tumor suppressor p53 further proposed the activation of several pathways due to the *SRC^V177M^* variant. The findings of our pedigree-based study contribute to the exploration of the genetic background of familial CRC and bring insights into the molecular basis of upregulated SRC activity and downstream pathways in colorectal carcinogenesis.

## 1. Introduction

Colorectal cancer (CRC) shows one of the largest proportions of familial cases among different malignancies, and thus it constitutes a promising target for next generation sequencing (NGS) as a tool for unravelling the underlying genetic alterations [1]. In addition to the established cancer predisposing genes, including the mismatch repair genes *MLH1, MSH2*, and *PMS2* as well as *APC, MUTYH,* and *SMAD4/BMPR1A*, recent sequencing studies have identified the *NTHL1*, *RNF43*, *POLE,* and *POLD1* genes as novel susceptibility genes underlying CRC inheritance [2,3,4,5,6,7,8]. Since germline variants in the described genes are considered to contribute to only 5–10% of all CRC cases, the remaining proportion of familial CRC, not linked to the discovered cancer predisposing genes, has to be further investigated [8,9,10].

In order to bring insight into the genetic background of unexplored familial CRC, we performed whole genome sequencing (WGS) in combination with integrative in silico analysis on a family presenting CRCs in three generations. Sequencing data were analyzed using the Familial Cancer Variant Prioritization Pipeline version 2 (FCVPPv2), developed by us, and implemented in the previous analysis of various familial malignancies, such as Hodgkin lymphoma and thyroid cancer [11,12,13]. The results converged to a few candidate genes which were further evaluated by additional in silico analysis.

## 2. Materials and Methods

### 2.1. Patient Samples & Ethical Permissions

**Figure 1:**
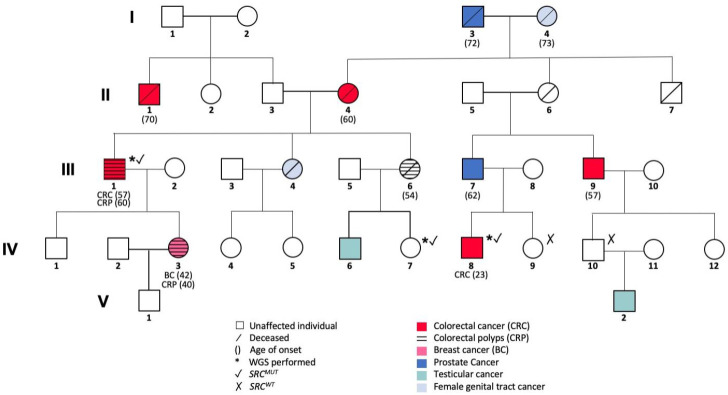
Pedigree of the studied colorectal cancer (CRC) affected family with the *SRC^V177M^* mutation.

The CRC-affected family of this study was recruited from Poland. The respective pedigree is shown in Figure 1, representing the CRC-affected members III-1 and IV-8 and the unaffected family member IV-7 that were included in our WGS analysis. Collection of blood samples and clinical information from subjects was undertaken with informed written consent in accordance with the tenets of the declaration of Helsinki.

The study was approved by the Bioethics Committee of the Pomeranian Medical Academy in Szczecin No: BN-001/174/05.

### 2.2. Whole Genome Sequencing and Variant Calling, Annotation and Filtering

Peripheral blood samples were collected from affected and unaffected family members who agreed to participate in the study as well as from the validation cohort. Genomic DNA was isolated using a modified Lahiri and Schnabel method [14]. WGS was performed using Illumina-based small read sequencing. After mapping to the reference human genome (assembly version Hs37d5) with BWA [15], duplicates were removed using Picard (http://broadinstitute.github.io/picard/ (accessed on 22 January 2021)).

Applying SAM tools [16] and Platypus [17], single nucleotide variants (SNVs) and small indels were detected, respectively. ANNOVAR [18], 1000 Genomes [19], dbSNP [20] and Exome Aggregation Consortium (ExAC) [21] were used for variant annotation. Variants with a quality score of ≥20 and a coverage score of ≥5×, SNVs passing the strand bias filter (a minimum one read support from both forward and reverse strand) and indels passing all the Platypus internal filters were further checked for minor allele frequencies (MAFs). With respect to the 1000 Genomes Project Phase 3, non-TCGA ExAC data [21], NHLBI-ESP6500 and local data sets, variants with a MAF ≤ 0.1% in the European population were selected for further analysis. A pairwise comparison of shared rare variants among cohort was performed to check for sample swaps and family relatedness.

### 2.3. Familial Segregation of the Cancer Predisposing Variant

The studied family shows aggregation of CRC and multiple other malignancies such as prostate, female genital tract, testicular, and breast cancer. In order to define familial segregation criteria for the pathogenic variant predisposing for cancer development in this family, the hereditary line of malignant diseases was retraced, assigning to each analyzed family member a probability of being a Mendelian case and carrier of the mutation (Figure 1).

The first case sequenced in this family (III-1) developed CRC as well as colorectal polyps (CRP) at the age of 57 and 60 years, respectively, and was thus considered as a carrier of the mutation. Tracing genetic cancer predisposition back to his CRC-affected mother (II-4) and further to his cancer-affected grandparents (I-3, I-4; prostate, female genital tract cancer, respectively), the cancer predisposing mutation might have been further inherited to his first cousin once removed (IV-8; via II-6 and III-7). Since this family member (IV-8) developed CRC at the young age of 23 years, he was regarded as the second case of the family and thus carrier of the mutation. On the other hand, the CRC-unaffected family member included in this study (IV-7) was 39 years at the time of recruitment. Her first-degree relatives were affected by cancer or CRP (IV-6, and III-6, respectively), suggesting that she might show the genotype without expressing the disease phenotype yet. Thus, she was considered as a possible carrier of the mutation.

The identified variants were filtered according to the described definitions of III-1 and IV-8 as cases and IV-7 as a possible carrier of the family, respectively summarized in Supplementary Table S1.

### 2.4. Evaluation of the Pathogenicity of Identified Variants Using FCVPPv2

Applying our in-house developed FCVPPv2, the cancer predisposing potential of coding variants was evaluated, including non-synonymous, stop-gain, small indels, and exonic variants of unknown classification.

Ranking all variants using the combined annotation dependent depletion tool (CADD) v1.3, only the top 10% of potentially deleterious variants represented by a PHRED-like (i.e., log_10_-derived) CADD score ≥ 10 were deduced for further analysis [22]. Since evolutionary conservation is regarded to correlate with the functional importance of a genomic position, conservational screening of variants was performed using the following scoring tools with respective cutoff values given in brackets: Genomic Evolutionary Rate Profiling (GERP ≥ 2.0), PhastCons (>0.3) and PhyloP score (≥3.0) [23,24]. In order to further assess the intolerance of genes against functional genetic variation, three intolerance scores (<0) based on allele frequency data from our in-house datasets, from ESP [25] and ExAC [26] were applied. Furthermore, intolerance screening of variants included the application of the Z-Score (>0) and pLI score (probability of being loss-of-function intolerant, ≥0.9), developed from ExAC consortium specifically for missense and loss-of-function variants, respectively. Next, the deleteriousness of non-synonymous and splice site SNVs was evaluated, using 10 different scoring systems and 2 meta-prediction tools derived from dbNSFP v3.0 (database for nonsynonymous SNPs’ functional predictions) [27]. In order to be further considered in the analysis, the variants should fulfill following filtering criteria: PHRED-like CADD-score of ≥10, ≥2 out of 3 conservational scores, ≥60% of 4 intolerance scores and ≥60% of 12 deleteriousness scores. The remaining top exonic candidates were assessed for allele frequencies in the non-Finnish European population using the latest version of gnomAD browser (https://gnomad.broadinstitute.org/ (accessed on 19 January 2020)) [28], for predicted cancer drivers by means of the Cancer Genome Interpreter (CGI, https://www.cancergenomeinterpreter.org/ (accessed on 20 January 2020)) [29] and for predicted functional effects of respective amino acid substitutions by Snap^2^ [30]. Conclusively, recent literature was checked for reported gene-cancer relations and potentially cancer-related protein functions of the top candidates.

### 2.5. Confirmation of Familial Segregation by Sanger Sequencing

Polymerase Chain Reaction (PCR) was performed with HotStarTaq DNA Polymerase (Qiagen, Hilden, DE, #203205) at an annealing temperature of 56 °C in order to amplify exon 5 of the *SRC* gene (ENST00000358208.4) from DNA of family members III-1, IV-7, IV-8, IV-9, and IV-10. The primers were designed with Primer3 v.0.4.0 (http://bioinfo.ut.ee/primer3-0.4.0/ (accessed on 11 February 2020)) as followed: *SRC* forward 5′-GGCTACATCCCCAGCAACTA-3′, reverse 5′-CCTCCCTACTCCACAAACCA-3′. PCR amplicons were validated by gel electrophoresis and purified with ExoSAP purification kit according to the manufacturer’s instruction. Sequencing reaction was performed using the BigDye Terminator v3.1 Ready Reaction Cycle Sequencing kit (Thermo Fisher Scientific, Waltham, MA, USA, #4337455), followed by manual analysis of the electrophoretic profiles of *SRC* sequences.

### 2.6. Screening of Familial CRC Index Cases and Healthy Individuals by Taqman Assay

The *SRC* variant was screened in 1690 familial CRC cases not related to the studied family and 1676 healthy elderly individuals, both from Poland, using a custom-made Taqman assay.

### 2.7. Plasmid Preparation and Cell Culture

pcDNA3-MTS-WT-c-Src-FLAG (#44652) was purchased from Addgene (Watertown, MA, USA) and used in functional experiments as the wild type *SRC* plasmid (*SRC^WT^*). The mutant *SRC* plasmid (*SRC^V177M^*) was created by using QuikChange II XL Site-Directed Mutagenesis Kit (Agilent Technologies, Santa Clara, CA, USA, #200521) and following primers designed based on Agilent QuikChange Primer Design (https://www.agilent.com/store/primerDesignProgram.jsp (accessed on 11 February 2020)): forward 5′-gtctcactttctcgcatgaggaaggtccctctc-3′, reverse 5′-gagagggaccttcctcatgcgagaaagtgagac-3′. After confirmation by Sanger sequencing, both plasmids were transformed into XL10-Gold Ultracompetent Cells (Agilent Technologies, Santa Clara, CA, USA, #200314) and plasmid extraction was performed using PureLink™ HiPure Plasmid Midiprep Kit (Thermo Fisher Scientific, Waltham, MA, USA, #K210004) according to manufacturer’s instructions.

Human colon cancer cell line HT-29 was a kind gift from Peter Krammer’s lab (DKFZ). HT-29 cells were cultured in RPMI and used for transfection of *pcDNA3* (HT29-*pcDNA3*), *SRC^WT^* (HT29-*SRC^WT^*), and *SRC^V177M^* (HT29-*SRC^V177M^*). Stably transfected pools of cells were selected by using G418.

### 2.8. Cell Proliferation Assays

HT-29 cells were seeded in 24-well plates and 24 h later transfected with either 150 ng of *SRC^WT^*, *SRC^V177M^* or *pcDNA3* vector as a negative control. After washing with PBS and trypsinizing the cells, viable cells were selected with trypan blue exclusion of dead cells and quantified by cell counting with the haemocytometer under a 10× objective at six different time points: day 0, 1, 2, 3, 4, and 5. Numbers of viable cells and respective proliferation curves were compared between HT29-*SRC^WT^* and HT29-*SRC^V177M^* cells.

### 2.9. Quantitative Polymerase Chain Reaction

RNA extraction from cells (HT29-*SRC^WT^*, HT29-*SRC^V177M^* and HT29-*pcDNA3*) was performed with Trizol and subsequent RNA purification with sodium acetate. ProtoScript First Strand cDNA Synthesis kit (New England Biolabs, Ipswich, MA, USA, #E6300S) was used for cDNA synthesis according to the manufacturer’s instructions. Quantitative Polymerase Chain Reaction (qPCR) was performed by means of QuantiFast^®^ SYBR^®^ Green PCR (Qiagen, Hilden, DE, Germany, #204054). The utilized primer pairs for SRC downstream targets (*paxillin*, *PXN; β-Catenin, CTNNB1; signal transducer and activator of transcription 3, STAT3; AKT*) and the housekeeping gene *HPRT* (*hypoxanthine phosphoribosyltransferase*) as a reference are summarized with respective primer sequences in Supplementary Table S2. Relative gene expression was calculated with the 2ΔCT method and compared between HT29-*SRC^WT^* and HT29-*SRC^V177M^* cells.

### 2.10. Western Blot

Protein lysates from HT29-*SRC^WT^*, HT29-*SRC^V177M^* and HT29-*pcDNA3* cells were prepared and quantified by means of Pierce™ BCA Protein Assay Kit (Thermo Fisher Scientific, Waltham, MA, USA, #23225). NuPAGE™ 4–12% Bis-Tris Protein Gels and the respective running buffer (Thermo Fisher Scientific, Waltham, MA, USA; #NP0321PK2, #NP0001) were used for separation of 20 μg of each protein sample. Blotted membranes were blocked with 2% milk for 1 h, incubated overnight at 4 °C with primary antibody dilutions and subsequently for 1 h at room temperature with the respective HRP-conjugated secondary antibody, diluted in 5% milk. Blots were developed using Amersham ECL Western Blotting Detection Kit (GE Healthcare, Chicago, IL, USA, #RPN2108). Glyceraldehyde 3-phosphate dehydrogenase (GAPDH) and β-Actin proteins were used for loading quantity control. All probed antibodies are summarized in Supplementary Table S3 with respective product details, dilution buffers, and dilution factors.

## 3. Results

### 3.1. Familial Cancer Variant Prioritization Pipeline Identifies a Novel Germline Variant in SRC Gene

**Figure 2:**
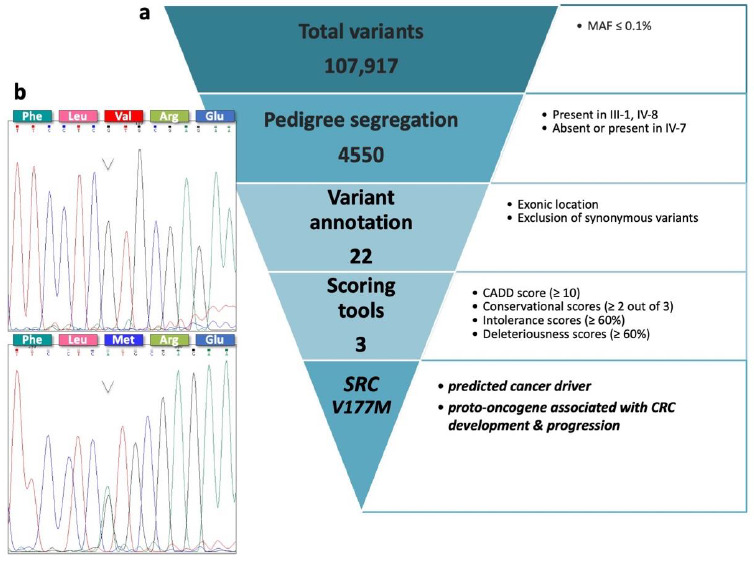
Prioritization of the missense variant in the *SRC* gene (V177M). (**a**) Flow chart depicting the filtering process of exonic variants according to the FCVPPv2. (**b**) Electropherograms representing the wild-type *SRC* sequence (upper panel) identified in family members IV-9 and IV-10 and the heterozygous *SRC^V177M^* variant (lower panel) identified in family members III-1, IV-7 and IV-8. The respective substitution Val → Met is displayed in the amino acid sequences.

In order to screen WGS data of the analyzed family members for cancer predisposing variants, we applied our in-house developed FCVPPv2 pipeline (Figure 2a). Filtering with a MAF ≤ 0.1% revealed a total number of 107,917 variants. By considering the familial segregation of the potentially cancer-causing mutation, 4550 variants were deduced for genomic location-based filtering. Most of these variants were annotated to affect intronic or intergenic regions, leaving 38 coding variants that were further analyzed. Removal of synonymous variants due to their potentially less deleterious nature resulted in 22 non-synonymous, stop-gain and variants of unknown classification. Application of the PHRED-like CADD score further narrowed down this number to 16 variants. Additionally, screening for evolutionary conservation, intolerance of the genes against functional genetic variation as well as predicted deleteriousness reduced the number of variants to 12, to 5 and ultimately to 3 final candidates, respectively: the non-synonymous variants in the *OGFOD2* (R11Q) and *SRC* genes (V177M) and the stop-gain variant in *ZNF408* gene (Q460X), which could only be annotated by 2 out of totally 12 deleteriousness scores due to its impact as a nonsense mutation (Table 1).

Checking the top-listed variants with the latest version of gnomAD, revealed allele frequencies of ≤0.1% in the Non-Finnish European population for all the variants [28]. On the other hand, CGI reported *SRC* as the only predicted cancer driver with an oncogenic function, whereas *OGFOD2* and *ZNF408* were annotated as passenger mutations [29]. These predictions were confirmed by the results of literature search stressing the carcinogenic potential of SRC.

### 3.2. Confirmation of Familial Segregation and Screening of a Large Cohort of Familial CRC Index Patients and Healthy Individuals

Targeted Sanger sequencing for exon 5 of the *SRC* gene confirmed pedigree segregation of the prioritized variant, showing the heterozygous mutation *SRC^V177M^* in two family members (III-1, IV-8) with CRC and in the possible carrier (IV-7) and the wild-type sequence in two family members without CRC, for whom the DNA samples were available and tested by Sanger sequencing (IV-9, IV-10, Figure 2b). Furthermore, targeted genotyping of 1690 unrelated familial CRC cases and 1676 healthy elderly individuals, both from Poland, using custom-made Taqman assay identified the *SRC^V177M^* variant in four additional index cases diagnosed at the ages of 48, 50, 60, and 65 years, respectively, and in three healthy individuals aged 63, 65, and 89 years, respectively (OR 1.65, 95%; CI 0.39–6.93, *p* = 0.49).

### 3.3. The Identified Variant Affects the Highly Conserved SH2 Domain of the SRC Protein

**Figure 3:**
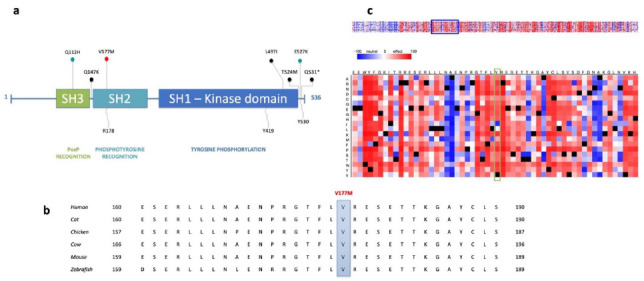
SRC domain structure and protein sequence highlighting the conserved and functionally important region affected by the missense variant V177M. (**a**) SRC protein domains are represented with respective domain functions: SH3 (84–145), SH2 (151–248), SH1 (270–523). Phosphorylation sites required for activation (R178 and Y419) and autoinhibition (Y530) are included. The identified germline variant affects the amino acid residue (V177M, red pin) directly adjacent to R178 within the SH2 domain, crucial for phosphotyrosine recognition. Additional *SRC* germline variants are indicated by blue pins (Q112H in CRC; E527K in thrombocytopenia, myelofibrosis, bleeding, bone pathologies [35]). Somatic mutations identified in CRC are represented by black pins: truncating mutation Q531 *, missense mutations extracted from cBioPortal (www.cbioportal.org (accessed on 20 March 2020)) using TCGA PanCancer data. (**b**) Extract of SRC protein sequence alignment downloaded from Ensemble (GRCh37/hg19) [34] for the following species: human (ENST00000373578.2), cow (ENSBTAT00000011767.3), mouse (ENSMUST00000029175.7), chicken (ENSGALT00000006127.2), cat (ENSFCAT00000006993.2), and zebrafish (ENSDART00000102843.4). As highlighted, the variant affects an amino acid residue identical in all sequences and thus highly conserved across the concerned species. (**c**) Predicted functional effects of amino acid substitutions are represented by the heat map extracted from Snap^2^, whereby the color red indicates a strong predicted effect, white an inconclusive prediction and blue a weak predicted effect. The position of the amino acid residue affected by the identified missense mutation (V177M) is highlighted with horizontal yellow box, showing an overall relatively high impact of potential substitutions at the respective position.

Analysis of the SRC protein sequence proposed a high functional impact of the affected position: First of all, the identified missense variant (V177M) alters an amino acid residue within the SH2 domain (pp. 151–248), a protein domain enabling physical interactions with phosphotyrosine-containing target peptides in the course of intracellular signaling cascades (Figure 3a). As part of several proteins including the Src, Fps, and Abl families, the SH2 domain shows high conservation, being identical in approximately 35% of all SH2 domains [32]. In particular, the universally conserved arginine residue R178 within the SH2 domain has been reported to play a central role in phosphotyrosine recognition and formation of electrostatic interactions [33]. Since the amino acid residue affected by the variant (V177M) is located directly adjacent to R178, the identified variant may have an impact on protein function and further protein–protein interactions. Alignment of SRC protein sequences of multiple species extracted from Ensembl (GRCh37/hg19), further revealed a high conservation of the whole protein (Supplementary Figure S1) and in particular of the affected region among all concerned species (Figure 3b) [34]. Similar results were obtained by Snap^2^ indicating an overall relatively high impact of potential substitutions at the respective position of the predicted amino acid change (Figure 3c).

Based on the established oncogenic role of SRC in general cancer development and in particular in CRC and on the described analysis results of the FCVPPv2, the identified *SRC^V177M^* variant was considered to bear pathogenic potential leading to its prioritization for functional validation.

### 3.4. Functional Validation of the Prioritized Variant in SRC Gene

#### 3.4.1. Enhanced Cell Proliferation of SRC^V177M^ Expressing CRC Cells in Vitro

**Figure 4:**
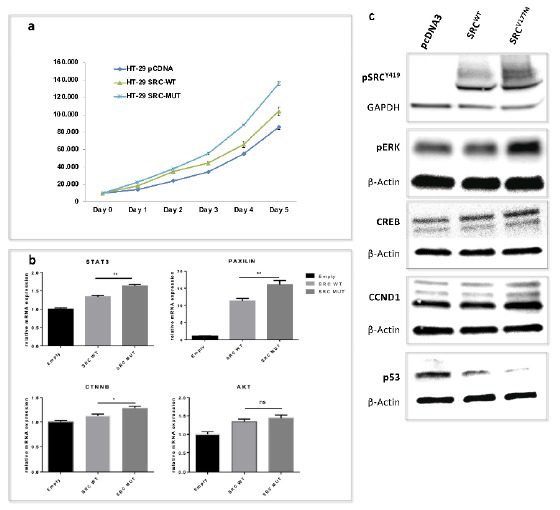
Impact of the *SRC^V177M^* variant on cell proliferation and key components of SRC signaling pathways. (**a**) Cell proliferation assays show significantly increased cell numbers of HT29-*SRC^V177M^* compared to HT29-*SRC^WT^* cells and the control. (**b**) qPCR results represent significantly increased mRNA levels of *PXN, CTNNB* and *STAT3* in HT29-*SRC^V177M^* cells. * *p* < 0.05; ** *p* < 0.01, ns—no significance. (**c**) Western blot results indicate enhanced protein expression of pSRC^Y419^, pERK, CREB and CCND1 as well as decreased p53 protein in HT29-*SRC^V177M^* cells.

In order to investigate the proliferative impact of the prioritized *SRC^V177M^* variant, cell proliferation assays were conducted at 6 different time points using HT-29 cells. Cells transfected with *SRC^V177M^* showed a significant increase in cell numbers compared to HT29-*SRC^WT^* cells starting from day 1 (*p* ≤ 0.0001). Cells transfected with *pcDNA3* showed the lowest cell numbers compared to both, HT29-*SRC^V177M^* and HT29-*SRC^WT^* cells, at all-time points (Figure 4a).

#### 3.4.2. Enhanced *STAT3*, *CTNNB*, and *PXN* Gene Transcription Induced by the *SRC^V177M^* Variant

In order to investigate the impact of the identified variant on pre-translational level, mRNA levels of potential target genes were quantified. Results of qPCR experiments showed significant upregulation of *CTNNB*, *STAT3* and *PXN* mRNA levels in HT29-*SRC^V177M^* compared to HT29-*SRC^WT^* cells (*CTNNB*: *p* < 0.05; *STAT3*, *PXN*: *p* < 0.01), whereas no significant difference could be observed for *AKT* mRNA levels. Thus, our experiments propose the involvement of the mutated SRC protein in pre-translational regulation of *CTNNB*, *STAT3* and *PXN* genes being associated with cell proliferation, invasion, and metastasis (Figure 4b).

#### 3.4.3. The *SRC^V177M^* Variant Leads to Increased SRC Phosphorylation at Y419, a Potential Marker for SRC Activity

In order to investigate the effect of the prioritized *SRC^V177M^* variant on SRC protein conformation and intrinsic kinase activity in vitro, HT-29 cells were transfected with the mutated plasmid and checked for phospho-SRC (pSRC) protein levels. Phosphorylation at the tyrosine residue 530 (pSRC^Y530^) has been reported to induce a closed SRC confirmation due to intramolecular binding of the respective phosphotyrosine to the SH2-domain. On the other hand, full activation of SRC requires an open protein conformation enabling autophosphorylation at position 419 (pSRC^Y419^) within the catalytic domain [36]. Western blot quantification of pSRC^Y419^ as a potential marker for activated SRC protein resulted in increased pSRC^Y419^ protein expression in HT29-*SRC^V177M^* cells compared to HT29-*SRC^WT^* cells. In this way, the *SRC^V177M^* variant enhanced the autophosphorylation and activation of SRC protein by potentially disrupting the pY530-SH2 domain interaction. Although HT-29 cells were shown to express SRC protein endogenously [37], the included control did not show detectable pSRC^Y419^ protein levels indicating the absence of the open and fully activated SRC protein conformation (Figure 4c).

#### 3.4.4. The *SRC^V177M^* Variant Affects pERK, CREB, CCND1, and p53 Protein Expression

With the aim of further validating the variant-induced upregulation of SRC activity and investigating the respective impact on colorectal carcinogenesis, key components of known SRC signaling pathways were checked for altered protein expression. Western Blot results revealed enhanced protein expression of phospho-ERK (extracellular signal-regulated kinase; pERK), CREB (CAMP responsive element binding protein), and CCND1 (cyclin D1) in HT29-*SRC^V177M^* compared to HT29-*SRC^WT^* cells. On the other hand, the tumor suppressor protein p53 showed decreased protein levels in HT29-*SRC^WT^* and, to an even greater extent, in HT29-*SRC^V177M^* cells compared to the control HT29-pcDNA3 (Figure 4c).

## 4. Discussion

By performing WGS and integrative in silico analysis on a CRC-affected family using our FCVPPv2, we were able to identify a novel germline variant in *SRC* gene (V177M) contributing to cancer predisposition. *SRC* is a commonly known proto-oncogene, the somatic mutations of which promote the development, progression and metastasis of various malignancies including colorectal, breast, prostate, ovarian, and testicular cancers [38,39,40]. However, the present results suggest that the identified *SRC^V177M^* variant may act as a germline CRC-predisposing variant. In contrast to numerous inactivating mutations in tumor suppressor genes, activating mutations contributing to familial cancer are rare and include the genes *RET, MET, KIT,* and *ALK* [41]. All of these encode kinases, which are activated by the predisposing mutations to different extent, which may be the mode of action of the present kinase, SRC. Non-complete penetrance of cancer and the diversity of cancers in the family may be explained by the observed moderate effect of the *SRC^V177M^* variant on CRC risk in the Polish population (OR 1.65). This suggests a polygenic mode of inheritance and additional mutations may be needed to express the cancer phenotype.

The oncogenic role of SRC has been elucidated on molecular basis, referring to cellular functions such as cell migration and invasion. One of the described underlying molecular mechanisms includes the focal adhesion-associated adaptor protein PXN: Docking at the phosphorylated tyrosine residue pY397 of Focal adhesion kinase (FAK), SRC can form the active FAK/SRC complex which further phosphorylates and associates with PXN and p130cas. Respective PXN/p130cas phosphotyrosines may then recruit Crk protein, resulting in cellular processes such as actin reorganization, cell spreading and migration [42,43,44]. In HT-29 cells, SRC-mediated increase of FAK, PXN and p130cas tyrosine phosphorylation and resulting cell migration enhancement has been induced by VEGFR-1 stimulation, implicating VEGF signaling upstream of the described molecular mechanisms [45].

**Figure 5:**
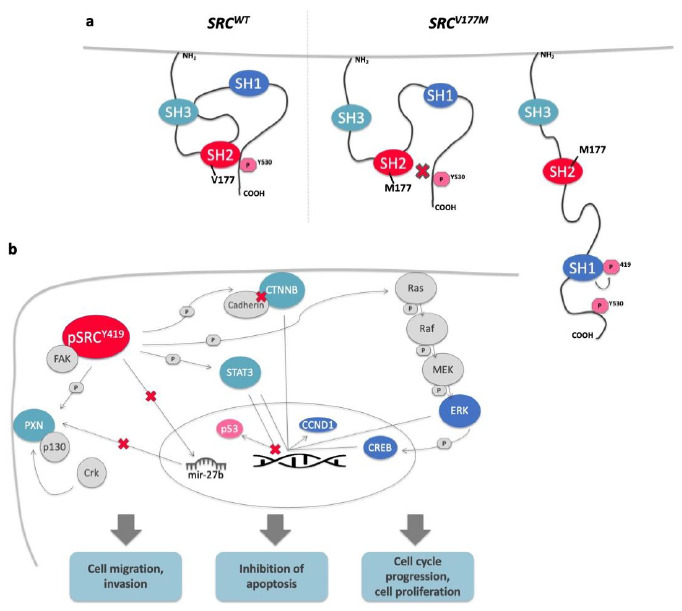
Molecular processes potentially induced by the *SRC^V177M^* variant. (**a**) The *SRC^V177M^* variant potentially induces disruption of the intramolecular pY530-SH2 domain binding leading to autophosphorylation at Y419 and full activation of the SRC protein. (**b**) Activated pSRC^Y419^ enhances cell migration, invasion, proliferation as well as cell cycle progression and inhibits apoptosis due to the illustrated cancer related pathways. The demonstrated activation of *CCND1* gene expression is representative of additional target genes of CTNNB, STAT3, ERK, and CREB, not illustrated in this figure. SRC target genes showing increased mRNA levels in HT29-*SRC^V177M^* cells were colored in light blue, whereas SRC target proteins overexpressed in HT29-*SRC^V177M^* cells were colored in dark blue. Observed suppression of protein expression due to the variant was represented by pink color. Arrows labeled with (P) are indicating phosphorylation, whereas arrows labeled with (X) are indicating an inhibiting impact.

In our experiments we showed that introduction of the *SRC^V177M^* variant resulted not only in upregulated protein expression of pSRC^Y419^, the fully activated SRC protein in open conformation (Figure 5a), but also in increased *PXN* mRNA levels. Thus, the mutated and activated SRC protein may affect *PXN* expression already at pre-translational level, potentially contributing to the described processes of cell migration. In addition to the described upregulation of *PXN* potentially contributing to invasive and migratory cell behavior, we observed increased *CTNNB* mRNA levels as a result of the introduced variant.

We also showed that *SRC^V177M^* upregulates *STAT3* at mRNA level. Although several studies have reported an increase in STAT3 transcriptional activity by SRC phosphorylation leading to gene expression of STAT3 target genes [46,47,48], little is known about the transcriptional regulation of *STAT3* itself, potentially involving SRC protein. A possible explanation approach may include the tumor suppressor protein p53, downregulated in our study by the *SRC^V177M^* variant. Since the well-established downstream effector miR-34a of p53 is known to inhibit the IL6R/STAT3/miR-34a feedback loop, a potential p53-mediated decrease of miR-34a may in turn lead to activation of IL6R/STAT3 signaling and thus CRC progression [49]. Even though the exact underlying mechanisms remain to be elucidated, our results indicate that the *SRC^V177M^* variant, and thus active pSRC^Y419^ may increase *STAT3* gene expression and may contribute to CRC. Several STAT3 target genes are known to play an important role in cell proliferation and apoptosis, such as *CCND1* [50]. In this study, we observed increased CCND1 protein levels due to the *SRC^V177M^* variant, which may lead to cell cycle progression and cell proliferation via the known SRC-STAT3-CCND1 association.

Additionally, *STAT3* has been reported to mediate SRC-induced transcriptional inhibition of the tumor suppressor p53 [51]. Since we observed decreased p53 protein levels upon *SRC^V177M^* variant introduction, these findings may also be explained by STAT3 as the mediating factor between activated pSRC^Y419^ and suppressed p53 expression, potentially resulting in inhibition of apoptosis. Taking the described activation of STAT3 by p53 downregulation into account [49], a reciprocal relation between STAT3 and p53 downstream of SRC may further be assumed. Thus, our results show conclusiveness based on an activating function of the studied *SRC^V177M^* variant.

In accordance with the described molecular functions, the affected STAT3 downstream targets CCND1 and p53 could be responsible for the observed increase of viable cell numbers of HT29-*SRC^V177M^* cells. Interestingly, overexpression of CCND1 may be traced back to further *SRC^V177M^* downstream effectors contributing to cell proliferation: 1. CTNNB activating gene transcription of Wnt target genes including *CCND1* [52] and 2. the MAPK/ERK pathway being required for *CCND1* transcription and assembly with CDK4/6 [53]. Since we observed increased phosphorylation of ERK protein in HT29-*SRC^V177M^* cells, our results indicate the potential activation of the proposed SRC-Ras-Raf-MEK-ERK1/2 pathway. Mediated by the Ras GTPase, SRC may induce the consecutive phosphorylation of the effector kinase Raf, MAP2K/MEK (Mitogen-activated protein kinase kinase) and MAPK/ERK, which is generally known to result in cell growth and proliferation [54]. In addition to *CCND1* transcription, ERK further affects the regulation of gene expression by phosphorylating CREB [54], which we also reported as overexpressed in HT29-*SRC^V177M^* cells.

The proposed association of SRC with activated PI3/AKT signaling, resulting in cellular processes such as cell growth, proliferation, and migration, is considered to rely on increased phosphorylation and activation of AKT protein [55,56]. Since our experiments investigated only *AKT* mRNA levels and did not reveal any differences between HT29-*SRC^V177M^* and HT29-*SRC^WT^* cells, our results indicate the independence of *AKT* gene expression regulation from the investigated mutation, not contradicting the current state of research.

Confirming the functional impact of the studied *SRC^V177M^* variant on key components of the described cancer related pathways (PXN, Wnt, STAT3, MAPK/ERK signaling, Figure 5b), we aim to confirm the postulated involvement of upregulated SRC activity in colorectal carcinogenesis and further to implicate these molecular mechanisms in cancer development of the studied family.

Although the *SRC* variant was reported in only 6 of 125,748 exomes of the gnomAD database, it was found in 4 additional index cases among 1690 tested Polish CRC families and in 3 out of 1676 controls, implying that it may be a moderate risk allele, explaining the non-complete penetrance of cancer in the family and also the diverse pattern of cancers which may be due to additional mutation(s).

Even though post-translational modifications of the SRC protein have been widely studied as the underlying cause of high SRC activity in cancer, less is known about activating variation of the *SRC* gene in human CRC besides the somatic truncating mutation *SRC^531^* [57,58]. By identifying a germline mutation of activating function, we were able to bring insight into the understanding of genetically determined upregulation of SRC activity in colorectal malignancies and to implement genetic *SRC* variation in familial CRC inheritance.

## Figures and Tables

**Table 1:** Exonic germline variants prioritized in the studied CRC family. Chromosomal positions, classifications, pedigree segregation, allele frequencies, PHRED-like combined annotation dependent depletion tool (CADD) scores, conservational scores and the percentage of positive intolerance and deleteriousness scores are summarized. CGI results, respective protein functions derived from GeneCards are included [31]. non-syn-non-synonymous; NFE-Non-Finnish European population; PP—predicted passenger; PD—predicted driver; OG—oncogene.

Gene Name	Chromosomal Position	Exonic Classification	Pedigree Segregation	Allele Frequency		CADD SCORE	Conservational Scores			Deleteriousness Scores * (%)	Intolerance Scores (%)	Amino Acid Change	CGI	Protein Function
				ExAC	gnomAD NFE		GERP++	PhyloP	PhastCons					
*OGFOD2*	12-123461223-G-A	nonsyn SNV	III1, IV8	6.2 × 10^−4^	7.1 × 10^−4^	24.8	5.67	6.69	1	91.67	60	R11Q	PP	Iron ion binding, oxidoreductase activity
*SRC*	20-36022656-G-A	nonsyn SNV	III1, IV7, IV8	1.8 × 10^−5^	3.9 × 10^−5^	26.8	4.88	6.64	1	91.67	100	V177M	PD, OG	Embryonic development, gene transcription, cell cycle progression, cell growth, adhesion, migration, transformation apoptosis, immune response
*ZNF408*	11-46726628-C-T	stop-gain SNV	III1, IV7, IV8	1.1 × 10^−4^	7.2 × 10^−5^	36	5.15	1.53	0.81	100 **	60	Q460X	PP	DNA binding protein, highly expressed in retina

* Deleteriousness scores: Following predictions were considered as favorable: SIFT—damaging; Polyphen2_HumDiv/HumVar—probably/possibly damaging; LRT—deleterious; MutationTaster—disease causing (automatic); MutationAssesor—high/medium; FATHMM—damaging; MetaSVM—damaging; MetaLR—damaging; VEST3 ≥ 0.5; PROVEAN—damaging; Reliability Index ≥ 5. ** Only 2 out of 12 deleteriousness scores were available for this variant.

## Data Availability

Unfortunately, for reasons of ethics and patient confidentiality, we are not able to provide the sequencing data into a public database. The data underlying the results presented in the study are available from the corresponding author or from Asta Försti (Email: a.foersti@kitz-heidelberg.de).

## Supplementary Materials

The following are available online at https://www.mdpi.com/article/10.3390/jpm11040262/s1, Table S1: Summary of family members WGS was performed on, including personal data and the consideration of being a carrier of the cancer-causing mutation; Table S2: Alphabetical list of qPCR primers with respective forward and reverse sequences; Table S3: Alphabetical list of primary and secondary antibodies with respective product details and dilution conditions; Figure S1: Alignment of multiple SRC protein sequences.
