# Supplementary material for: A Novel Low-Risk Germline Variant in the SH2 Domain of the SRC Gene Affects Multiple Pathways in Familial Colorectal Cancer
Source: J Pers Med. 2021 Apr 1;11(4):262. doi: 10.3390/jpm11040262 (PMC8066297; doi:10.3390/jpm11040262)
Supplement: Supplementary file 1 [file jpm-11-00262-s001.pdf]

Supplementary Materials

Supplementary Table S1 Summary of family members WGS was performed on, including personal data and the consideration of being a carrier of the cancer-causing mutation. CRC – colorectal cancer, CRP – colorectal polyps.

Classification	ID	Sex	Age at recruitment	Diagnosis	Age of onset	Considered as a carrier of the mutation?
Cases	III-1	male	77	CRC, CRP	57, 60	Yes
	IV-8	male	54	CRC	23	Yes
Possible carrier	IV-7	female	40	-	-	Yes/No

Supplementary Table S2. Alphabetical list of qPCR primers with respective forward and reverse sequences.

Gene	Forward primer sequence	Reverse primer sequence
AKT	Quantitect primer assay purchased from Qiagen	
CTNNB	CACAAGCAGAGTGCTGAAGGTG	GATTCCTGAGAGTCCAAAGACAG
HPRT	Quantitect primer assay purchased from Qiagen	
PXN	CTGATGGCTTCGCTGTCGGATT	GCTTGTTCAGGTCAGACTGCAG
STAT3	CTTTGAGACCGAGGTGTATCACC	GGTCAGCATGTTGTACCACAGG

Supplementary Table S3. Alphabetical list of primary and secondary antibodies with respective product details and dilution conditions.

PRIMARY ANTIBODIES

Protein target	Host	Catalog Number	Company	Dilution buffer	Dilution factor
β-Actin	Rabbit	ab119716	Abcam	5% milk in TBST	1:4000
CCND1	Rabbit	ab134175	Abcam	5% milk in TBST	1:3000
CREB	Mouse	86B10	Cell Signaling Technology	5% milk in TBST	1:1000
pERK	Rabbit	PA5-37823	ThermoFisher	5% milk in TBST	1:500
GAPDH	Mouse	CB1001	Millipore	5% milk in TBST	1:3000
p53	Mouse	1C12	Cell Signaling Technology	5% milk in TBST	1:1000
pSRC(Y419)	Rabbit	ab185617	Abcam	4% BSA in TBST	1:5000

SECONDARY ANTIBODIES

Species reactivity	Class	Catalog number	Company	Dilution buffer	Dilution factor
Mouse	Horse	7076s	Cell Signaling Technology	5% milk in TBST	1:5000
Rabbit	Goat	7074s	Cell Signaling Technology	5% milk in TBST	1:5000

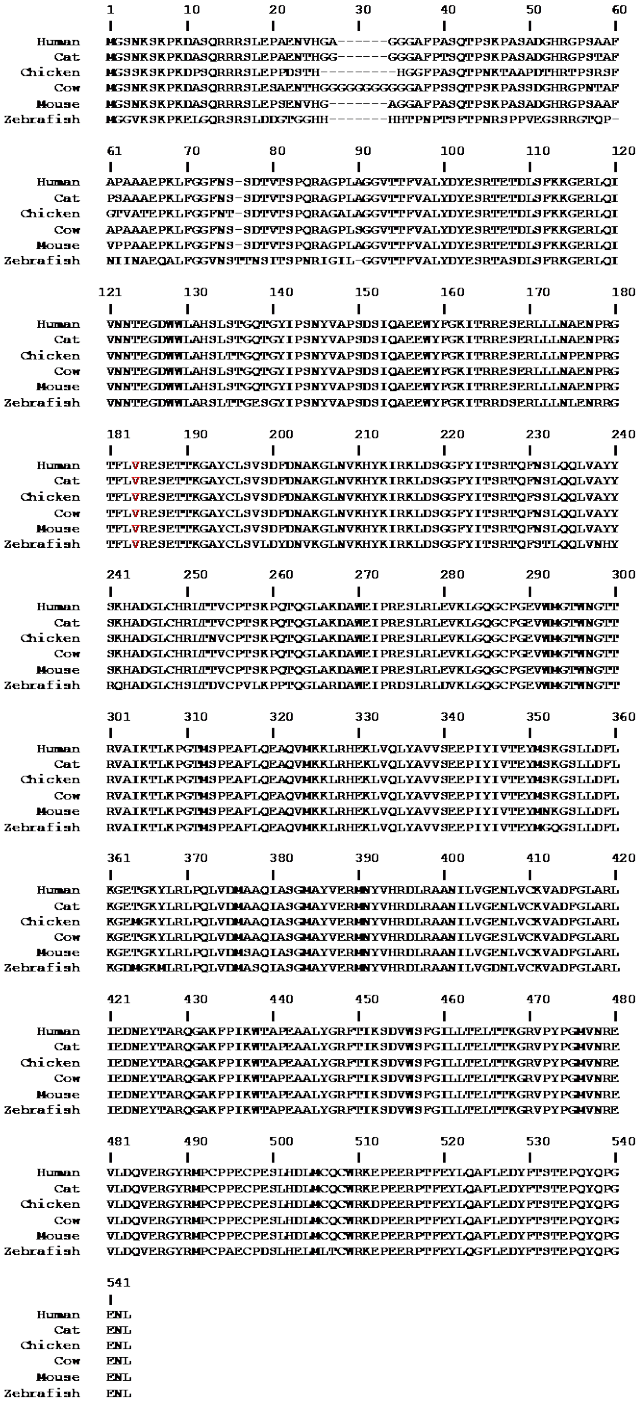

Supplementary Figure S1. Alignment of multiple SRC protein sequences. SRC protein sequences were downloaded from Ensembl (GRCh37/hg19) for human (ENST00000373578.2), cow (ENSBTAT00000011767.3), mouse (ENSMUST00000029175.7), chicken (ENSGALT00000006127.2), cat (ENSFCAT00000006993.2) and zebra fish (ENSDART00000102843.4) and respectively aligned. Sequence alignment indicates an overall strong conservation of SRC across the species. The amino acid residue affected by the *SRC*^{V177M} variant is colored in red.
